# Enhanced IL-1β Release Following NLRP3 and AIM2 Inflammasome Stimulation Is Linked to mtROS in Airway Macrophages in Pulmonary Fibrosis

**DOI:** 10.3389/fimmu.2021.661811

**Published:** 2021-06-15

**Authors:** Athina Trachalaki, Eliza Tsitoura, Semeli Mastrodimou, Rachele Invernizzi, Eirini Vasarmidi, Eleni Bibaki, Nikolaos Tzanakis, Philip L. Molyneaux, Toby M. Maher, Katerina Antoniou

**Affiliations:** ^1^ Laboratory of Molecular and Cellular Pneumonology, Respiratory Medicine Department, School of Medicine, University of Crete, Heraklion, Greece; ^2^ National Heart and Lung Institute, Imperial College London, London, United Kingdom; ^3^ Royal Brompton Hospital, London, United Kingdom

**Keywords:** IPF – idiopathic pulmonary fibrosis, ILD, NLRP3, AIM2, NLRC4, mtROS, mitochondrial reactive oxygen species, microbiome

## Abstract

Fibrotic Interstitial lung diseases (ILDs) are complex disorders of variable clinical behaviour. The majority of them cause significant morbidity, whilst Idiopathic Pulmonary Fibrosis (IPF) is recognised as the most relentless. NLRP3, AIM2, and NLRC4 inflammasomes are multiprotein complexes driving IL-1β release; a proinflammatory and profibrotic cytokine. Several pathogenetic factors associated with IPF are identified as inflammasome activators, including increases in mtROS and bacterial burden. Mitochondrial oxidation and alterations in bacterial burden in IPF and other ILDs may lead to augmented inflammasome activity in airway macrophages (AMs). IPF (n=14), non-IPF-ILDs (n=12) patients and healthy subjects (n=12) were prospectively recruited and AMs were isolated from bronchoalveolar lavage. IL-1β release resulting from NLRP3, AIM2 and NLRC4 inflammasomes stimulation in AMs were determined and baseline levels of mitochondrial ROS and microbial burden were also measured. Our results showed that NLRP3 was more inducible in IPF and other ILDs compared to controls. Additionally, following AIM2 activation IL-1β release was significantly higher in IPF compared to controls, whereas similar trends were observed in Non-IPF-ILDs. NLRC4 activation was similar across groups. mtROS was significantly associated with heightened NLRP3 and AIM2 activation, and mitochondrial antioxidant treatment limited inflammasome activation. Importantly, microbial burden was linked to baseline IL-1β release and *AIM2* and *IL-18* relative expression independently of mtROS. In conclusion, the above findings suggested a link between the overactivation of NLRP3 and AIM2 inflammasomes, driven by mitochondrial oxidation, in the pathogenesis of lung fibrosis while changes in the microbiota may prime the inflammasome in the lungs.

## Introduction

Interstitial Lung Disease (ILD) is a broad term used to describe multiple complex disorders with different aetiologies and disease behaviours. ILDs are pulmonary fibrotic disorders that affect the lung alveoli causing distraction of the lung parenchyma. The classic paradigm is Idiopathic Pulmonary Fibrosis (IPF), a devastating chronic lung disease of unknown aetiology (1). An interplay of age dependent deregulation of host defence pathways, immune cell homeostasis and microbiome balance is currently speculated to be the underlying cause of the disease (2). Other ILDs are considered to be driven mostly through inflammatory pathways (2).

In the last decade, the landscape of the IPF behaviour has changed with the discovery of two novel antifibrotic drugs (3–5). Of note, the two antifibrotic treatments are similarly effective in other progressive ILDs (6–8). This suggests progressive fibrosis arises from common underlying mechanisms, despite the initial cause, but the exact pathways remain to be identified (9).

Macrophages and monocytes are recognised as drivers of the balance between tissue repair and fibrosis (10). Recent studies in ILD patients have characterised Airway Macrophages (AM) subpopulations which may contribute to different subtypes of pulmonary fibrosis (11). Macrophages, as a source of chemokines and other inflammatory mediators, are responsible for the initial cellular response to injury (12) and can initiate or exacerbate fibrosis (10).

The inflammasome is one central cellular mechanism for chemokine release in macrophages (13). Inflammasomes are cytosolic multiprotein complexes that act as innate immune system sensors. NLRP3, the most studied inflammasome, is activated by a variety of stimuli including ATP, nigericin and ROS (14). Other inflammasomes are more specific and activated by merely one stimulus; NLRC4 by cytosolic Flagellin and AIM2 by cytosolic double stranded DNA (dsDNA) (15). Upon triggering, Inflammasomes cleave pro-IL-1β and pro-IL-18 to their active forms mainly through caspase-1 (15). IL-1β is a potent pro-inflammatory cytokine that can initiate and amplify lung inflammation and has been associated with acute lung injury and fibrosis (16–18). Inflammasome activation has been associated with silicosis, asbestosis and pulmonary fibrosis, mostly in mice models (19–21).

Mitochondria as drivers of metabolic cascades are increasingly recognised as controllers of macrophage activation status (22–25). Mitochondrial reactive oxygen species (mtROS) and oxidised mitochondrial DNA (mtDNA) are both critical for NLRP3 activation (25, 26). It is established that mitochondria homeostasis is defective in aged and IPF individuals (27, 28) and their dysfunction contribute directly to fibrosis in mouse models (29). Our group and others have recently demonstrated that mitochondria in AMs are more oxidised in IPF and other ILDs compared to healthy individuals (30–32).

Microbiota signalling has been shown to alter mitochondrial metabolism and activate the inflammasome (33–35). In IPF, the microbial burden is increased, and its composition is altered (36, 37). Similarly in Chronic Hypersensitivity Pneumonitis (CHP), bacterial burden is higher, albeit at lower levels compared to IPF (38). These alterations have been linked to host immune response transcriptional changes (39) and variable cytokine secretion (40). Of note, streptococcal infection leads to acute exacerbation of lung fibrosis in mice through AIM2 inflammasome activation (41) while inflammasome activation was dysregulated in BAL cells from ILD patients (42).

In this study we hypothesised that alterations in the lung microenvironment including microbiome changes and mitochondrial dysfunction in AMs could drive excessive inflammasome activation with possible implications in the pathogenesis of ILD disease. As such, we sought to determine whether increased mtROS and microbial burden were associated with inflammasome activity in AMs from ILDs, including IPF.

## Materials and Methods

### Patients and Inclusion Criteria

Thirty-six (34) patients were prospectively enrolled at the Respiratory Medicine Department at the University Hospital of Heraklion, Crete, between June 2017 to June 2019.

Twelve (12) healthy controls, and twenty-four (24) ILD patients [fourteen (14) patients with IPF and twelve (12) non-IPF patients] were included. Patients with a recent infection (1 month prior to bronchoscopy) were excluded from the study. ILD patients were treatment naïve. All patients underwent bronchoscopy and Bronchoalveolar Lavage Fluid (BALF) was obtained as part of the diagnostic algorithm.

IPF patients: The diagnosis of IPF was based on either ATS/ERS criteria or the Fleischer Society criteria after Multidisciplinary discussion (22).

Non-IPF patients: Patients with fibrotic Interstitial Lung Diseases as assessed by the presence of reticulation and traction bronchiectasis on High-Resolution Computed Tomography were included. This category included eight (8) patients with fibrotic chronic hypersensitivity pneumonitis (CHP), one (1) with asbestosis related ILD, one (1) with Idiopathic pneumonia with autoimmune features (IPAF) and two (2) with unclassifiable ILD. Any patients with autoimmune diseases treated with immunosuppressive agents were excluded as immunomodulatory treatment could affect inflammatory responses. All patients were evaluated within one month from bronchoscopy, with Pulmonary Lung Function tests.

Control group: Control subjects were either patients undergoing bronchoscopy for the investigation of haemoptysis, without any overt pulmonary comorbidities, with normal bronchoscopy findings and cytology results or healthy volunteers.

Patient demographics, smocking status, and pulmonary function tests (PFTs) were prospectively collected and are summarised in **Table 1**. Since controls were healthy Pulmonary Function Test (PFTs) were not performed.

**Table 1:** Patient characteristics.

	Control	IPF	Non-IPF	
n	12	14	12	
Age	56.9 ± 14	74.9 ± 6	69 ± 11	P<0001
Male/Female	8/4	12/2	8/4	P ns
Smoking status				P ns
Never smokers	1	2	4	
Smokers	11	13	8	
Pack years	38.1 ± .33.8	40.2 ± 17.4	37.5 ± 19.5	P ns
FVC%		87 ± 21	90 ± 26	P ns
FEV1%		95 ± 19	95 ± 27	P ns
DLCO%		65 ± 21	58 ± 22	P ns
TLC%		81 ± 17	82.8 ± 26	P ns
Macrophages%	93 ± 5	88 ± 5	s89 ± 8	P ns
Lymphocytes%	6 ± 4	6 ± 5	4 ± 3	P ns
Neutrophils%	2 ± 1.5	3 ± 2	4 ± 4	P ns
Eosinophils%	0.4 ± 0.2	1.4 ± 1.3	1.4 ± 1.8	P ns

ns, non significant.

PFTs. Lung volumes (forced expiratory volume in one second – FEV1, forced vital capacity – FVC), and diffusion capacity (DLco, corrected for haemoglobin) were measured using the computerised system (Jaeger 2.12; MasterLab, Würzburg, Germany). Predicted values were obtained from the standardised lung function testing of the European Coal and Steel Community, Luxembourg (1993).

### AM Isolation and Culture

Freshly isolated BALF cells were obtained as previously described (30). BALF contains a diverse population of primarily macrophages and variable levels of neutrophils, lymphocytes and eosinophils. To allow macrophage enrichment, BAL cells were cultured for 1 hour prior to experimentation. For each experimental condition 0.5x10^6^ BALF cells were allowed to attach for 1 hour in 24 well plates in DMEM(Biosera) growth media supplemented with 2% FCS (Biosera) and 1x concentration of penicillin-streptomycin (from 100x concentrated solution, Biosera) in a humidified incubator at 37°C containing 5% CO2 at a concentration of 10^6^ cells/ml, with subsequent washes to remove non-adherent cells. The remaining attached cell population comprised mainly of macrophages and monocytes from the alveolar space.

### Inflammasomes Activation

Human macrophages are known to require a two-step mechanism to activate NLRP3; an evolutionary process preventing uncontrolled NLRP3 activation and IL-1β release. The primary TLR-mediated signal activates NF-kappaB (NF-κB) which results in NLRP3, ASC, pro-IL-1β and pro-IL-18 transcription. The second signal results in proteolytic cleavage of IL-1β and IL-18 by caspase-1 (43).

Here, AMs were primed with 10ng/ml LPS (Liposacccharide E.Coli 0111:B4 strain, Invivogen) to upregulate inflammasome related genes. For NLRP3 activation, cells were primed with LPS for 2 hours and subsequently stimulated with 5 mM ATP(Sigma) for 30 minutes. For the activation of AIM2 and NLRC4 inflammasomes, cells were primed with LPS for 1 hour, followed by transfection with 2 μg/ml dsDNA(naked Poly(dA:dT), InvivoGen) or 0.1ug/ml Ultrapure Flagellin from S. Typhimirium, (InvivoGen) respectively, using Lipofectamine 2000 (Life Technologies) for 4 hours. Inflammasome-specific activation was confirmed using the selective NLRP3 inhibitor MCC950(Cayman) at 1μM, or the caspase-1 inhibitor for the NLRC4 and AIM2 inflammasome (non-selective global inflammasome inhibitor) at 10uM final concentrations, for 1 hour prior to stimulation. The simulation protocol is summarised in **Supplementary Figure 1**.

For the study of mtROS effect on NLRP3 activation, cells were treated with the selective mitochondrial antioxidant agent MitoTempo (Sigma) at a final concentration of 100μM for 1 hour prior to the addition of ATP.

Supernatants and cell lysates were collected after the appropriate stimulation time. ELISA immunoblot was used to quantify IL-1β, as a surrogate marker of inflammasome activation.

### Flow Cytometry and mtROS Determination

Mitochondrial ROS was measured by MitoSOX™Red (Invitrogen) staining. 0.5x10^6^ freshly isolated BALF cells resuspended in RPMI-1640, supplemented with 2% FCS, were stained with MitoSOX Red at a final concentration of 5 μM and CD45-FITC for 10 minutes at 37°C. For MitoSOX staining quantification, AMs populations were selected according to high forward and side scatter and CD45 (FSC^high^SSC^high^CD45^+^) as previously described (30). Identical cell samples were independently stained with Propidium Iodide (PI) at a final concentration of 1ng/ml, for 5 minutes immediately before flow cytometry analysis, for the detection of necrotic/apoptotic cells. The percentage of MitoSOX positive cells was determined by the percent of cells showing FL-2 fluorescence higher than the unstained control, followed by subtraction of the PI positive percentage of cells. Relative mean fluorescence intensity (MFI) was calculated by normalizing the MFI of the FL-2 channel/MitoSOX positive cells by the MFI of the FL-2 channel of the unstained cells since patient samples displayed wide ranges of autofluorescence. (Data were acquired from Beckman Coulter flow cytometer and analysed with FlowJo 8.7).

### Bacterial DNA Extraction and 16S rRNA Gene qPCR

Bacterial DNA extraction and 16S rRNA gene qPCR was performed for quantification of bacterial burden as previously described (36).

### Total RNA Extraction and mRNA Expression

1-1.5x10^6^ BALF cells were centrifuged and cell pellets were homogenised in TriReagentTM (MBL) for total RNA extraction, followed by storage at -80°C. Total RNA extraction, cDNA synthesis and real-time PCR were performed as previously described (24). GAPDH levels were used as endogenous control for the normalization of mRNA expression levels in BAL samples. Primer sequences are shown in **Supplementary Table 1**.

### Statistical Analysis

Data were analysed using SPSS 25 (IBM) software and graphs were produced using GraphPad Prism 8. Comparisons were made with paired or unpaired student’s *t-*test, when appropriate. All data were expressed as means with interquartile range unless stated otherwise. Receiver operating characteristics (ROC) curve analysis was used to select an optimal cut point for mtROS. Spearman’s correlation coefficient (*r*) analysis measured the association between two variables. A *p* value less than 0.05 was considered statistically significant (*p<0.05, **p<0.01, ***p<0.001).

### Ethics Approval and Patient Consent

The study was approved by the Ethics Committees of the University Hospital of Heraklion (IRB number: 5889). Approval for the study in the UK was obtained from the local research ethics committee (15/SC/0101 and 15-LO-1399). All patients provided written informed consent.

## Results

### BALF Baseline IL-1β and Inflammasome Components Gene Expression Is Similar in ILDs and Healthy Subjects

**Figure 1:**
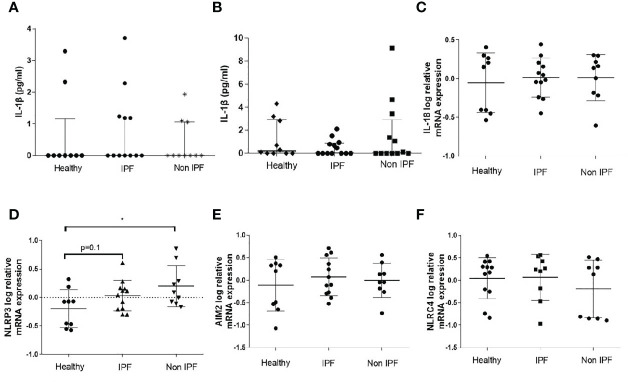
Baseline characterization of the Inflammasome in BALF cells. **(A)** IL-1β protein concentration in BAL fluid as measured by ELISA and **(B)** IL-1β protein release by unstimulated AMs (5-hour culture). Data represented as median with interquartile range Mann-Whitney test. Relative log mRNA expression in unstimulated BALF cells **(C)** *IL18* **(D)** *NLRP3*, **(E)** *AIM2*, **(F)** *NLRC4*. Data represented as mean ± SD, t-test. *p<0.05.

Initially, the baseline IL-1β levels were measured in the BAL Fluid (BALF), as a marker of inflammasome pre-activation in the lung microenvironment. Median basal IL-1β concentration in the BALF did not differ significantly between the ILDs and control groups, while some sporadic IL-1β release was detectable in all three groups (**Figure 1A**). IL-1β secretion by unstimulated freshly isolated AMs was also measured and no differences were observed among the groups (**Figure 1B**). Furthermore, we assessed the mRNA expression of genes encoding core inflammasome components *NLRP3*, *NLRC4*, and *AIM2* as well as the gene encoding *IL-18* in BALF cells (**Figures 1C–F**). *NLRP3* relative mRNA expression was significantly elevated in Non-IPF ILDs (p=0.03) and tended to be elevated in IPF as well (p=0.1). *AIM2*, *NLCR4* and *IL-18* expression was similar between groups.

### AMs in ILDs Exhibit Enhanced NLRP3 Inflammasome Activation

Typically, monocytes/macrophages require two independent simulation signals to secrete IL-1β *in vitro*; an NF-κB priming step (signal 1), such as LPS or other TLR4 agonists, which leads to the upregulation of inflammasome pathway related genes, followed by a stimulation with a DAMP or PAMP which acts as a second stimulus (signal 2) and leads to robust IL-1β release. Signal 1 alone is known to activate the NLRP3 in a non-canonical way in human myeloid cells (44, 45).

**Figure 2:**
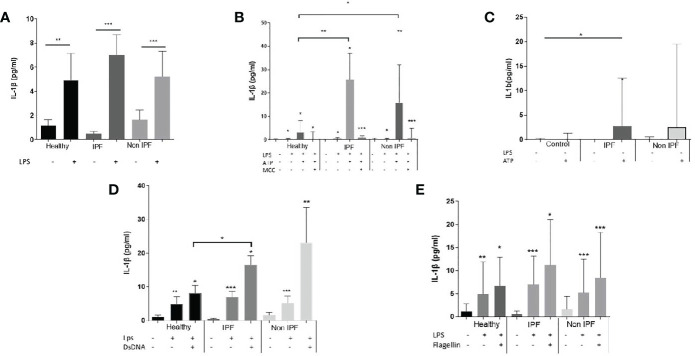
Assay of inflammasomes activation in Airway Macrophages (AMs). **(A)** AMs treated for 5 hours with LPS **(B)** Activation of NLRP3 upon priming with LPS for 2 hours and ATP for 30minutes. Cells were also treated in presence of the Selective NLRP3 inhibitor MCC950 to show specificity of the NLRP3 activation, **(C)** AMs cultured for 2 hours in serum and treated for 30 minutes with ATP without priming with LPS **(D)** AIM2 inflammasome activation: 1 hour of LPS priming followed by 4 hours dsDNA transfection, **(E)** NLRC4 inflammasome activation: 1 hour of LPS priming followed by 4 hours flagellin transfection. For with-in group analysis one-sided paired Wilcoxin test was used. For between group analysis Mann-Whitney test was used. Data presented as median with interquartile range, *p<0.05, **p<0.005, ***p<0.0001.

In this study, for NLRP3 inflammasome activation, AMs were primed with LPS followed by ATP stimulation. Treatment with LPS resulted in a significant release of IL-1β by AMs from all groups (**Figure 2A**), although no differences between groups were noted (**Figure 2A**). Upon NLRP3 activation, IPF and non-IPF AMs produced excessive IL-1β compared to controls (p=0.0004 and 0.007 respectively, **Figure 2B**). Between IPF and Non-IPF patients, NLRP3 activity was similar. This effect was NLRP3 specific, as it was abrogated by MCC950 treatment (**Figure 2B**), a novel specific NLRP3 inhibitor (46).

To determine whether ILD derived AMs were pre-primed by an NF-κB related stimuli in the lungs, we also treated cells with ATP (with no proceeding signal 1). Generally, ATP alone resulted in muted responses compared to LPS/ATP. However, in IPF the addition of ATP was sufficient to generate IL-1β release, compared to healthy controls (p=0.04, **Figure 2C**). Similar trends were noted in non-IPF ILDs (p=0.1, **Figure 2C**). When all ILDs were combined, ATP resulted in increased NLRP3 activation compared to controls (p=0.04).

### AIM2 and NLRC4 Activation

For AIM2 activation, cells were primed with LPS followed by transfection with dsDNA. In all three groups the addition of dsDNA resulted in significant IL-1β release (**Figure 2D**). IL-1β production following dsDNA stimulation was significantly elevated in IPF compared to healthy controls (p=0.04) whilst a similar trend was observed for the Non-IPF patients (p=0.09) (**Figure 2D**).

It was recently suggested that in human myeloid cells, in contrast to mice, dsDNA activates the cGAS-STING pathway. This action potentiates lysosomal damage and in turn activates the NLRP3 rather than the AIM2 inflammasome (47). To address this possibility, cells were treated with dsDNA in the presence of MCC950 and a significant inhibition of IL-1β release was observed (**Supplementary Figure 2A**). To test further the above hypothesis of STING-mediated lysosomal damage, cells were treated with chloroquine, a lysosomal acidification inhibitor that blocks lysosomal induced cell death. Chloroquine inhibited dsDNA-mediated inflammasome activation (**Supplementary Figure 1A**).

For the activation of NLRC4, cells were primed with LPS, followed by flagellin transfection. The activation of NLRC4 inflammasome was similar across groups (**Figure 2E**). To confirm the specificity of NLRC4 activation in AMs, both the pan-inflammasome Caspase-1 inhibitor and the NLRP3 specific inhibitor-MCC950 were tested. NLRC4 stimulation resulted in IL-1β release in a caspase-1 dependent and NLRP3-independent manner (**Supplementary Figure 2B**).

### NLRP3 and AIM2 Activation Is Associated With Mitochondrial Oxidation and Can Be Inhibited by Antioxidant Treatment

**Figure 3:**
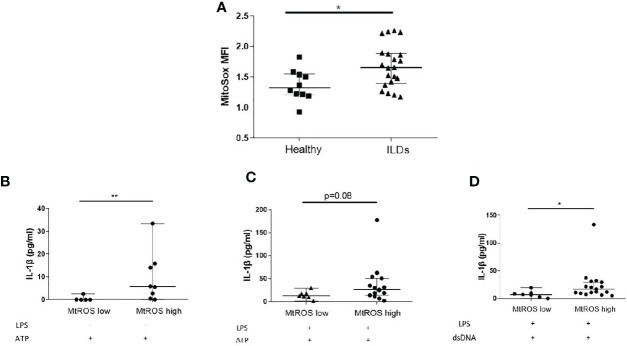
Mitochondrial Oxidation status and the inflammasome. **(A)** MtROS Mean Florescence Intensity Index (MFI) as assessed by flowcytometry in healthy and ILD patients. ILD Patients were categorised according to MtROS MFI using the cut-off value of 1.5(generated by ROC curve). Comparisons on inflammasome activation were made in MtROS high and low groups **(B)** NLRP3 activation without LPS priming, **(C)** NLRP3 activation with LPS priming, **(D)** AIM2 activation (LPS priming followed by dsDNA transfection). Data presented as median with interquartile range, *p<0.05, **p<0.005, Mann-Whitney test.

MtROS is widely recognised as an inducer of inflammasome activation and it was previously shown that mtROS is elevated in IPF and Non-IPF AMs (30, 32). It was therefore hypothesised that elevated mtROS could be associated with higher NLRP3 inflammasome activity in ILDs. The levels of mtROS in fresh untreated AMs was measured by flow cytometry using MitoSOX™red. MtROS was higher in ILD-AMs (IPF and Non-IPF) compared to controls (p=0.03, **Figure 3A**).

Using ROC curves the optimal cut-off value for mtROS level was determined and ILD-AMs samples were stratified according to high or low mtROS. AMs exhibiting high mtROS showed enhanced NLRP3 activation in the absence of LPS priming (p=0.004, **Figure 3B**) and there was also a trend for greater NLRP3 activation in LPS-primed AMs (p=0.08) (**Figure 3C**). AIM2 activation was likewise significantly heightened in AMs with high mtROS (p= 0.02) (**Figure 3D**).

**Figure 4:**
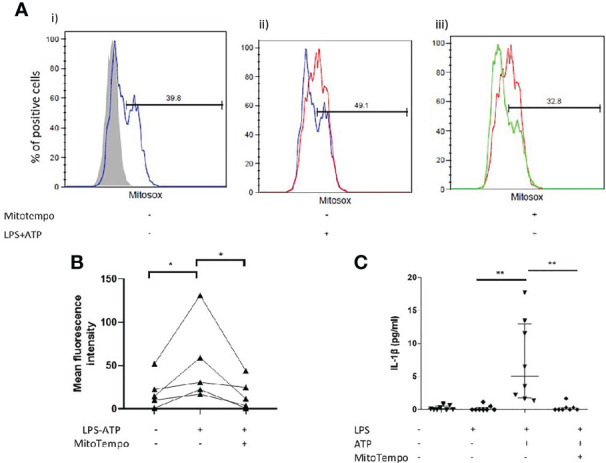
Mitochondrial antioxidant treatment effect on inflammasome activation. AMs were treated with MitoTempo (100μM), a mitochondrial antioxidant for 1 hour prior to the addition of ATP. Cells were subsequently treated with ATP for 30minutes. **(A)** Representative histograms of freshly isolated AMs from ILD subjects, labelled with CD45-FITC and MitoSOX Red analysed by flowcytometry. AMs were selected and percentage of MitoSOX positive cells and mean fluorescence intensities were analysed relative to CD45-FITC labelled populations (grey histograms). AMs were either left untreated(i), treated with (ii) LPS/ATP for NLRP3 activation or (iii) MitoTempo and LPS/ATP. **(B)** MitoSox (assessed by flow-cytometry) and **(C)** IL-1β release measured at baseline, after NLRP3 activation and after NLRP3 activation in the presence of MitoTempo (100μM), Data presented as median with interquartile range, *p<0.05 **p<0.005, Paired Wilcoxon test.

Interestingly, *in vitro* stimulation of AMs with LPS and ATP coincided with a burst of mtROS (**Figures 4A, B**). We subsequently sought to determine whether antioxidant treatment could inhibit IL-1β release by AMs. Treatment with mitoTempo a mitochondria-targeting antioxidant significantly inhibited mtROS accumulation (**Figures 4A, B**) and blocked NLRP3 activation, as assessed by IL-1β release (**Figure 4C**).

### Microbial Burden Is Associated With Baseline *AIM2* and *IL-18* Gene Expression and Il-1β Release

**Figure 5:**
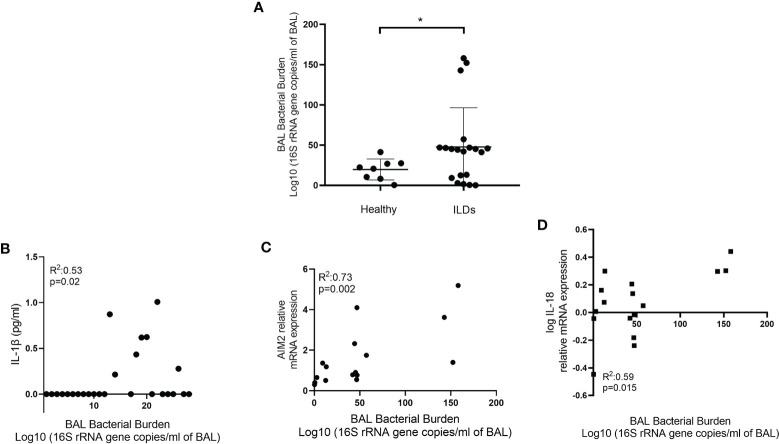
Bacterial burden and the inflammasome. **(A)** Bacterial burden in bronchoalveolar lavage (BAL) of healthy and ILD subjects. Bacterial burden calculated by qPCR and expressed as log10 (16S rRNA gene copies/ml of BAL). Mann-Whitney test. Data are presented as median and interquartile range, *p<0.05. Illustrating correlation between bacterial burden and **(B)** IL-1β release from AMs (Spearman correlation) and **(C)** *AIM2* and **(D)** *IL-18* log relative mRNA expression (Pearson Correlation).

In IPF, it is established that microbial burden is increased and this correlates with disease progression (36, 37). In CHP, microbial burden is also elevated compared to health but at lower levels compared to IPF (38). We hypothesised that microbiota fluctuations in the lung microenvironment could be priming the inflammasomes. BALF microbial burden, as assessed by 16S rRNA gene copies, increased in ILDs relative to healthy individuals (p=0.03) (**Figure 5A**). Furthermore, 16S rRNA gene copies significantly correlated with IL-1β secretion in unstimulated AMs (R^2^:0.53; p=0.02)(**Figure 5B**) and *AIM2* (R^2^: 0.68 p=0.004, **Figure 5C**) and *IL-18* (R^2^: 0.59 p=0.015, **Figure 5D**) relative mRNA expression. There was no association between bacterial burden and mtROS.

## Discussion

In this study we demonstrated that AMs in pulmonary fibrosis secrete abundantly IL-1β following stimulation of the NLRP3 and AIM2 inflammasomes compared to healthy subjects. Several factors that could be involved in inflammasomes stimulation have been previously identified in IPF, including increased mtROS and increased microbiome burden. This study showed that higher mtROS was associated with pronounced NLRP3 and AIM2 activation which could be inhibited by antioxidant therapy. Furthermore, BALF microbial burden correlated with baseline IL-1β production, *AIM2* and *IL-18* relative mRNA expression.

The overactivation of the inflammasome has multiple implications in health and disease. Several animal model studies have suggested that NLRP3 activation and subsequent IL-1β release can induce acute lung injury and fibrosis (48–50). In mice models the NLRP3/IL1-b axis is required for the development of bleomycin induced fibrosis (49). In silicosis there is a clear link between NLRP3 and the development of the disease (51). Furthermore, in mice age-dependent mitochondrial dysfunction results in enhanced NLRP3 activation and lung fibrosis (20). In this study we provide evidence of an implication of NLRP3 and AIM2 inflammasome in patients with lung fibrosis.

ROS generation by the mitochondria is a well-known trigger of the NLRP3 with mitochondrial death and mitochondrial DNA acting as activators (25). Mitochondrial dysfunction has emerged as a driver of IPF pathogenesis. Mitochondria in IPF AMs have morphological defects and are oxidised (30). Similarly, mtROS is elevated in other ILDs (32). To the best of our knowledge this is the first study to show that in ILD-AMs NLRP3 and AIM2 inflammasome are hyper-inducible and this is associated with mitochondrial oxidation, a hallmark of IPF pathogenesis. In agreement with previous studies (26), we showed that mtROS is crucial for NLRP3 inflammasome activation in patient derived-AMs, since antioxidant treatment inhibits IL-1β release.

It is established that a priming step is required for the activation of the inflammasome which results in the overexpression of several inflammasome components (43). The second signal results in the release of active IL-1β. ROS can act as a direct NLRP3 activator (52), while other studies have suggested that ROS could also exert its role at the priming step (24). Of note, treatment with ATP without LPS pre-stimulation resulted in pronounced IL-1β release in ILDs compared to controls. Furthermore, inflammasome was more inducible in patients with higher mtROS levels, in the absence of LPS priming. This implies that mitochondrial oxidation may be priming the inflammasome in lung fibrosis.

Mitochondria are in the core of metabolic switches in macrophages fighting bacterial infections (53) and a burst of mtROS is crucial for their antibacterial responses (51). By contrast, mitochondrial damage results in deregulated and diminished antioxidant responses to bacteria (54). It is recognised that microbiota are altered in IPF (55, 56). In this study, microbial burden, which is known to be increased in ILDs, was not associated with mtROS, indicating that microbiota changes were not likely the cause of the observed mitochondrial oxidation. The microbial burden was however associated with baseline IL-1β release as well as *AIM2* and *IL-18* mRNA expression in lung fibrosis.

AIM2 inflammasome was recently linked to the pathogenesis of lung fibrosis and progression. Notably, AIM2 is overexpressed in IPF-AMs and this is related to increased Drosha ribonuclease III (DROSHA), a class 2 ribonuclease III enzyme expression (57). Interestingly, a previous study showed that in IPF, peripheral mononuclear cells stimulated to activate the AIM2 released high concentrations of pro-fibrotic mediators and most importantly IL- 1α (58). Of particular interest, GLUT-1 dependent glycolysis promotes exacerbation of lung fibrosis during S. pneumoniae infection *via* AIM2 activation (41) and several studies have suggested a relative abundance of Streptococcus genera in IPF (37, 38, 56). A novel finding of our study is that AIM2 activation is increased in IPF and tended to be higher in other fibrotic ILDs. AIM2 activation was enhanced in patients with higher mtROS and an increase in the bacterial burden was associated with baseline *AIM2* expression

For years, AIM2 was recognised as the central DNA-responding inflammasome. Recent evidence suggests that AIM2 might not be functional in human immune cells in contrast to murine models (47). Researchers showed that cytosolic DNA causes lysosomal damage and activation of the NLRP3 inflammasome through the STING mediated cell death pathway. In our experiments, addition of dsDNA resulted in significant release of IL-1β in all groups. Inhibition of NLRP3 with MCC950, a specific NLRP3 inhibitor, resulted in partial reduction of IL-1β release, as such suggesting that dsDNA activation is to a degree NLRP3-dependent. We also showed that higher mtROS was associated with higher IL-1β production following dsDNA treatment, a result which could be driven by NLRP3 activation rather than AIM2.

In contrast to NLRP3 and AIM2, NLRC4 activation was similar across groups. NLRC4 activation was caspase-1 dependent and NLRP3 independent. A previous transcriptional study in IPF, showed that NLRC4 expression is increased in the peripheral blood and highly associated with increased microbial burden in the lungs (39). Here we focused on AMs and failed to notice overexpression or overactivation of the NLRC4 inflammasome either at baseline or upon stimulation.

Our study sheds light into the pathogenesis of acute exacerbations, a well-recognised complication of IPF and other ILDs (59, 60), characterised by rapid deterioration and death. The pathogenesis of acute exacerbations is not well characterised but is thought to resemble acute lung injury in response to infections or sterile insults (60, 61). Microbiota changes have been implicated in the pathogenesis of IPF exacerbations (37, 56, 62). It is well established that dysregulated NLRP3 inflammasome activity results in uncontrolled inflammation and can cause acute lung injury and fibrosis (18, 50). Intriguingly, a transcriptomic study identified NLRP3 and IL-1β among the top up-regulated genes in AE-IPF (63).

This study indicates that in ILDs NLRP3 and AIM2 can be overactivated. Basal *AIM2* and *IL-18* mRNA expression and IL-1β production was correlated with the bacterial burden in the BALF. Although, it is difficult to prove a direct causal relationship between microbial burden and inflammasome activation, we speculated that microbiota changes prime the inflammasome in the lungs. It is established that microbiota dysbiosis influence systematic immune responses (64) and gut-microbiota changes shape cytokine release from leukocytes (65). More specifically in IPF, disruption of the lung microbiome was associated with variable cytokine production leading to lung inflammation and fibrosis progression (40). Targeting NLRP3, AIM2 or their ultimate effector, IL-1β and IL-18 (66) may prove a novel treatment for ILD exacerbations, which are still considered lethal in most cases.

The main limitation of this study is related to the small number of patients recruited, as differences among different types of lung fibrosis were not established. However, even in this small cohort, we were able to identify changes in lung fibrosis compared to health. Furthermore, different macrophage/monocyte populations exist in the lungs especially following a fibrotic insult (67). It is likely that one or more subpopulations drive the observed excessive inflammasome activation. As such, separation of the different AM populations and subsequent stimulations might be informative of the role of each subpopulation in the disease pathogenesis. One further limitation of our study is the measurement of IL-1β alone and not IL-18 as a marker of inflammasome stimulation. We opted to measure only IL-1β as it is considered a robust marker of inflammasome activation and has generally been associated with initiation and progression of fibrosis. Importantly, IL-1β is not constitutively expressed under homeostasis (68) and has detrimental effects on epithelial cells if over-released (69). Finally, although inflammasome activation in lung fibrosis was increased, we cannot determine whether this plays a causal role in disease progression, nor can we exclude that other factors may relate to the increased inflammasome activation observed here.

In conclusion, this is the first study to show that NLRP3 and AIM2 activation is heightened in ILD-AMs and this is linked to mitochondrial oxidation. Microbial burden is associated to some extent with pre-activation of the inflammasome. Our findings provide evidence of excessive AM-inflammatory response upon activation which could contribute to the progression of fibrosis. Targeting the inflammasome pathway may prove a novel strategy for ILD treatment or a rescue therapy during acute exacerbations of the disease.

## Data Availability Statement

The raw data supporting the conclusions of this article will be made available by the authors, without undue reservation.

## Ethics Statement

The study was approved by the Ethics Committees of the University Hospital of Heraklion (IRB number: 5889). Approval for the study in the UK was obtained from the local research ethics committee (15/SC/0101 and 15-LO-1399). The patients/participants provided their written informed consent to participate in this study.

## Author Contributions

Conception and design: AT, ET, KA. Analysis interpretation and drafting the manuscript: AT, ET, KA. TM, PM. Technical support and interpretation of the data: AT, ET, SM, RI, EV, EB. All authors critically revised the manuscript and take responsibility for the data presented. All authors contributed to the article and approved the submitted version.

## Funding

This study was supported by a Hellenic Thoracic Society Research Award (AT, KA HTS 2017). AT is the recipient of an ERS fellowship STFR (STRTF201904-00633).

## Conflict of Interest

The authors declare that the research was conducted in the absence of any commercial or financial relationships that could be construed as a potential conflict of interest

## Abbreviations

AECIIs, alveolar epithelial cells; AIM2, Absent in melanoma 2; AMs, Airway Macrophages; ARDS, Acute Respiratory Distress Syndrome; CHP, chronic hypersensitivity pneumonitis; BALF, Bronchoalveolar Lavage Fluid; dsDNA, double stranded DNA; IPF, Idiopathic Pulmonary Fibrosis; LPS, lipopolysaccharide; NLRC4, NLR Family CARD Domain Containing 4; NLRP3, NOD-, leucine rich region- and pyrin domain-containing-3; NLR, NOD-Like receptor; mtROS, Mitochondrial Reactive Oxygen Species; mtDNA, mitochondrial DNA; ROS, Reactive Oxygen Species.
