# Supplementary figures and images for: Enhanced IL-1β Release Following NLRP3 and AIM2 Inflammasome Stimulation Is Linked to mtROS in Airway Macrophages in Pulmonary Fibrosis
Source: Front Immunol. 2021 Jun 15;12:661811. doi: 10.3389/fimmu.2021.661811 (PMC8248801; doi:10.3389/fimmu.2021.661811)

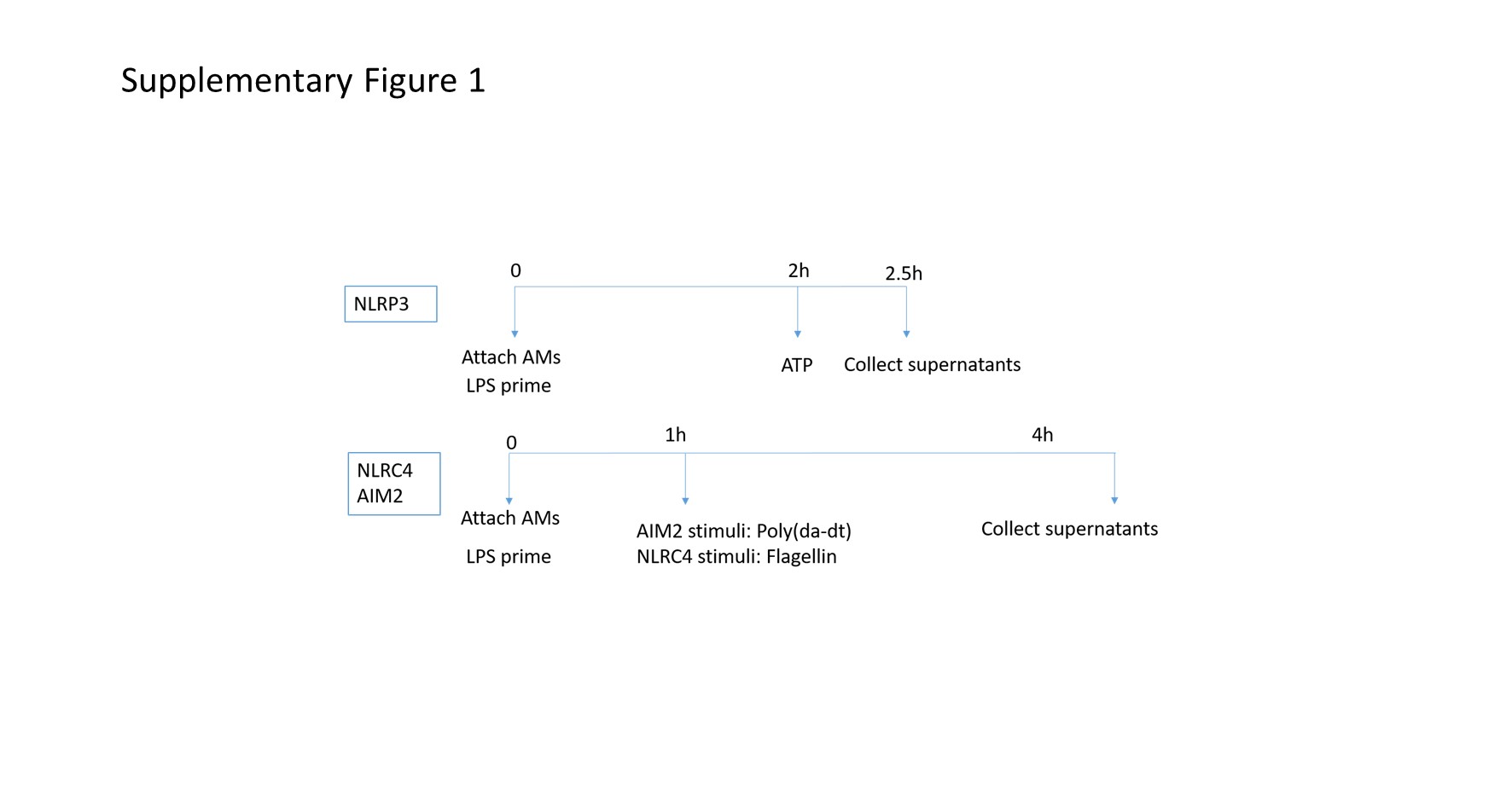

Supplement: Supplementary file 1 [file Image_1.jpeg]

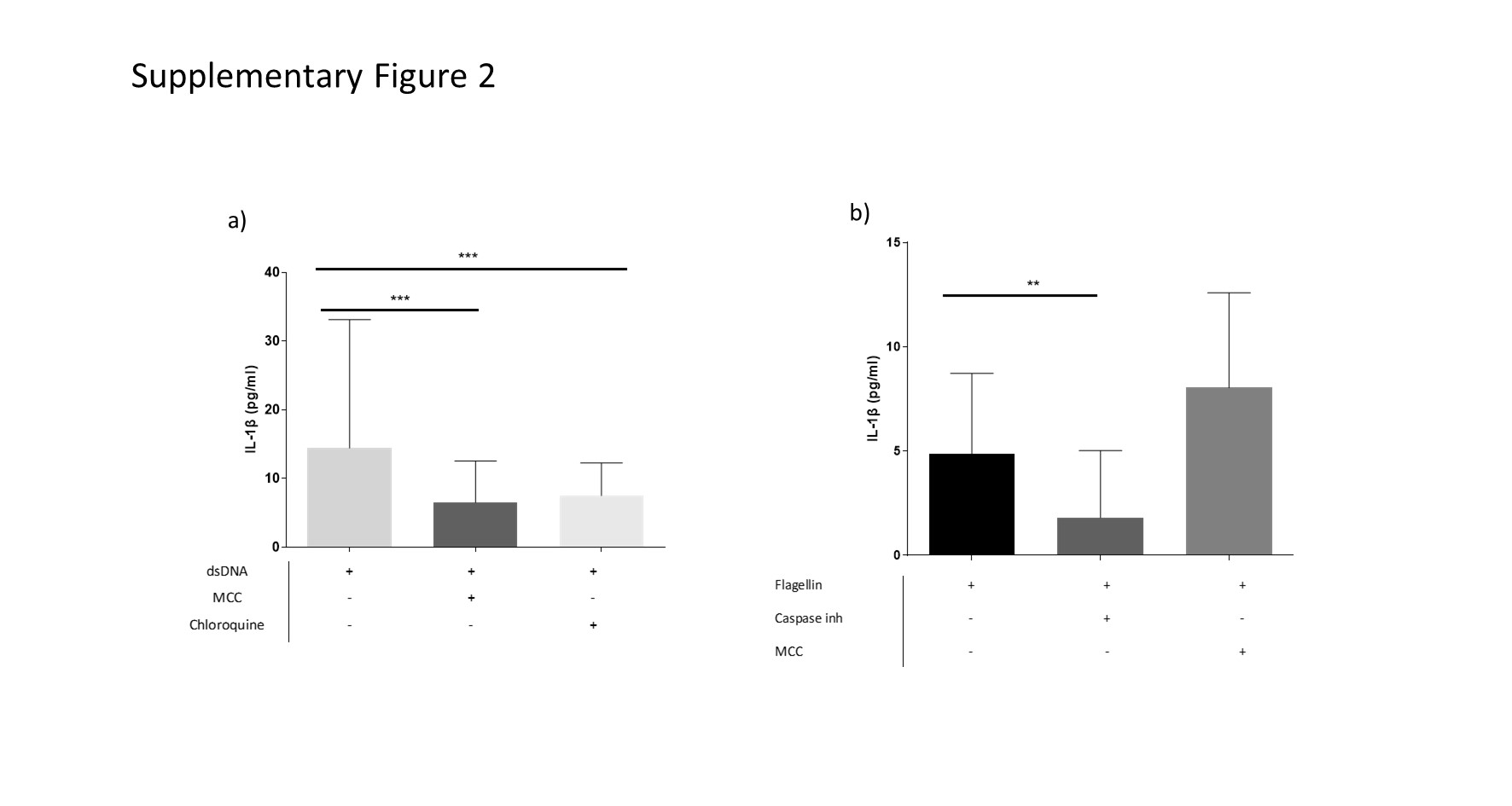

Supplement: Supplementary file 2 [file Image_2.jpeg]
