# Supplementary material for: Enhanced IL-1β Release Following NLRP3 and AIM2 Inflammasome Stimulation Is Linked to mtROS in Airway Macrophages in Pulmonary Fibrosis
Source: Front Immunol. 2021 Jun 15;12:661811. doi: 10.3389/fimmu.2021.661811 (PMC8248801; doi:10.3389/fimmu.2021.661811)
Supplement: Supplementary file 3 [file DataSheet_1.docx]

Supplementary Material

# Supplementary methods

**BAL cell isolation and determination of cellular composition**

Bronchoalveolar Lavage (BAL) was obtained as previously reported (1). In brief, a flexible bronchoscope was wedged into a sub-segmental bronchus of a predetermined region of interest based on radiographical findings. A BAL technique was performed by instilling a total of 180 ml of normal saline in 60-mL aliquots, each retrieved by low suction.

The BAL samples were subsequently kept on ice and were processed within 2 hours of collection. Samples were filtered through sterile 70nm cell strainers (BD) and centrifuged at 1500rpm for 5 minutes at 4^ο^C. Cell pellets were washed and re-suspended with cold PBS. Total cell count and cell viability were subsequently assessed using Trypan blue (ICN). Differential cell population count was analysed following May-Grunewald-Giemsa staining as previously described (2).

**Reagents:**

Ultrapure LPS (E. coli O111:B4), dsDNA-Poly(dA:dT) and purified S. typhimurium Flagellin was from Invivogen, ATP and mitoTEMPO was from Sigma-Aldrich. MCC950 was purchased from Cayman chemical and caspase-1 inhibitor from Calbiochem.

For the immunoblot analysis anti-mouse IL-1b (12426S, Cell Signaling Technologies) was used. Inflammasomes activation was assessed by the IL-1b release, using ELISA (ThermoFischer)

# Supplementary Figures and Tables

**Supplementary Table 1.** Primer sequences for qPCR.

| **Gene name** | **Primer sequences** |
| --- | --- |
| NLRP3 | F: CACCTGTTGTGCAATCTGAAG |
|  | R: GCAAGATCCTGACAACATGC |
| AIM2 | F: TGGTTTGTTTGTAGTCCAGAAGG |
|  | R: CCTCGTTTCTAACCCCCAGT |
| NLRC4 | F: CCTGAGCAGCCTGTTGAAA |
|  | R: AAGTTTTTCAGAGGGTTCTTTCC |
| GAPDH | F: AGCCACATCGCTCAGACAC |
|  | R: GCCCAATACGACCAAATCC |
| For MT-DNA quantification |  |
| MT-ND1 (MRC complex I) | F: AACCTCTCCACCCTTATCACAA |
|  | R: TCATATTATGGCCAAGGGTCA |

*NLRP3 Nod-like receptor protein 3, AIM2 Absent in melanoma 2, NLRC4 NLR Family CARD Domain Containing 4, GAPDH glyceraldehyde-3-phosphate dehydrogenase, MT-ND1 mitochondrial encoded NADH dehydrogenase 1,*

Supplementary Figure 1. Representative stimulation timeline

Supplementary Figure 2.

1. AMs from the same ILD patients were pre-stimulated with LPS and then treated with dsDNA,
2. or pretreated with LPS and MCC950 or c) Chloroquine before being treated with dsDNA before the addition of flagellin MCC: MCC950 is a selective NLRP3 inhibitor, Caspase inh: non-selective Caspace-1 inhibitor. The experiment was performed in more than five independent experiments. Data presented as median with interquartile range **p<0.005, *** p<0.000, Paired Wilcoxon test was performed

**
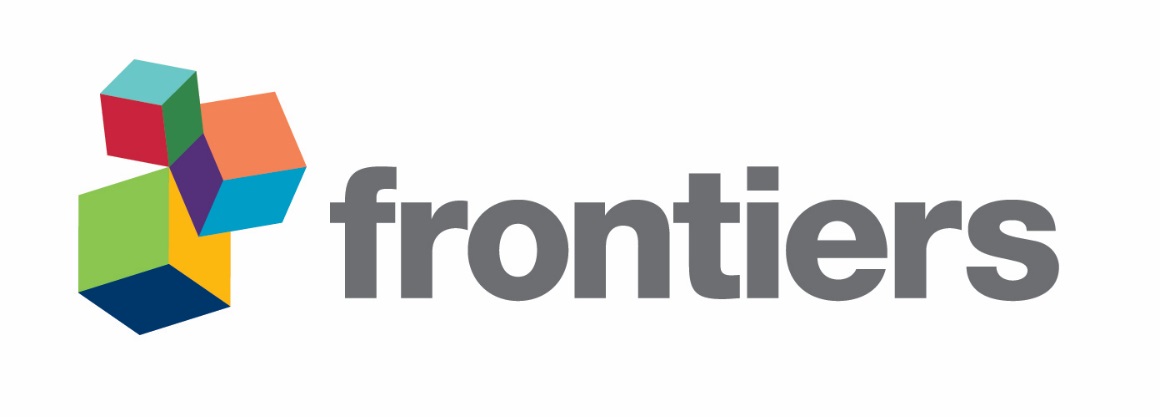
**

1. Samara KD, Trachalaki A, Tsitoura E, Koutsopoulos AV, Lagoudaki ED, Lasithiotaki I, et al. Upregulation of citrullination pathway: From Autoimmune to Idiopathic Lung Fibrosis. Respiratory research. 2017;18(1):218.

2. Tsitoura E, Wells AU, Karagiannis K, Lasithiotaki I, Vasarmidi E, Bibaki E, et al. MiR-185/AKT and miR-29a/collagen 1a pathways are activated in IPF BAL cells. Oncotarget. 2016;7(46):74569-81.
